# Prevalence of Undiagnosed Hypertension and Associated Factors among Adults in Mizan-Aman Town, Bench Sheko Zone, Southwest Ethiopia: A Community-Based Cross-Sectional Study

**DOI:** 10.1155/2023/2746284

**Published:** 2023-07-10

**Authors:** Sebsibe Elias, Teshome Kabeta Dadi

**Affiliations:** ^1^Public Health Department, College of Health Sciences, Mizan-Aman College of Health Science, Mizan-Aman, Ethiopia; ^2^Department of Epidemiology, Faculty of Public Health, Institute of Health, Jimma University, Jimma, Ethiopia

## Abstract

**Objectives:**

This study aimed to assess the prevalence of undiagnosed hypertension and associated factors among people aged 18 years and above in Mizan-Aman town of Bench Sheko Zone in Southwest Ethiopia. *Study Design*. A community-based cross-sectional study design was carried out among people aged 18 years old and above from April 1 to 30, 2021, in Mizan Aman town.

**Methods:**

A community-based cross-sectional study design was carried out among people aged 18 years old and above from April 1 to 30, 2021, in Mizan Aman town. Seven hundred fifty-nine subjects were selected by the multistage sampling technique. A structured pretested STEPwise questionnaire was used to interview the participants. Data entry and analysis were done using EpiData 3.1 and SPSS version 25 statistical software, respectively. Descriptive analysis was undertaken, and the results were presented using frequency tables, graphs, and statistical summaries. The dependent variable has a dichotomized response of yes and no, and hence binary logistic regression was used to predict a dependent variable based on independent variables, and predictors having *p* ≤ 0.25 on the bivariable analysis were considered as candidates for the multivariable analysis. Odds ratios with their 95% confidence intervals were calculated to measure the strength of association, and finally a *p* value <0.05 was considered statistically significant.

**Result:**

The prevalence of undiagnosed hypertension was 14.8% with 95% CI [12.3–15.6]. Older age (AOR = 3.1, 95% CI [1.5–6.5]), male (AOR = 2.2, 95% CI [1.3–3.9]), low physical activity (AOR = 3.9, 95% CI [1.8–8.3]), low consumption of fruit and vegetable (AOR = 4.5, 95% CI [2.4–8.8]), and higher BMI (AOR = 2.7, 95% CI [1.6–4.6]) were significantly associated with undiagnosed hypertension.

**Conclusion:**

The current study outlined that the prevalence of undiagnosed hypertension was high in the study area. In addition, most of the risk factors identified were modifiable, and hence community-based preventive approaches like lifestyle modification, increasing awareness, and strengthening routine screening at primary health service facilities resulted in a substantial change in tackling the burden effectively.

## 1. Background

Worldwide levels of undiagnosed hypertension (HTN) represent the global public health crisis. It affected around 22% of people aged 18 years and over and is responsible for an estimated 9.4 million deaths per year globally [1, 2]. Approximately 11 million U.S. adults with a usual source of health care have undiagnosed hypertension, placing them at increased risk for cardiovascular events [3]. In South-East Asian countries, more than 50% of people with hypertension remain undiagnosed [4]. The prevalence of undiagnosed hypertension was found to be 30% in sub-Saharan Africa (SSA) [5].

The study conducted in Dabat, Northern Ethiopia, reveals that the largest proportion (83.4%) of patients had not been diagnosed and/or treated for HTN [6]. The prevalence of undiagnosed HTN was 13.3% in Gulele sub-city, Addis Ababa, Ethiopia [7]. Another study conducted in Hawela Tula sub-city, Hawassa, Southern Ethiopia, reveals that the prevalence of undiagnosed hypertension was 12.3% [8].

Signs and symptoms of HTN are often undetectable during the early stages, and hence many people with the disease are left undiagnosed [9]. The preventable and modifiable factors for high blood pressure include behavioral risk factors, such as weight problems, excessive nutritional salt intake, low nutritional consumption of calcium and potassium, alcohol consumption, psychosocial stress, low tiers of physical activity, and non-behavioral risk factors like family history of high blood pressure, age, and sex. These risk factors result in various long-term disease processes, ending in high mortality rates attributable to stroke, heart attack, tobacco- and nutrition-induced cancers, and obstructive lung diseases [10–13]. However, diagnosis and treatment along with lifestyle modification are essential for the management of HTN. In developing countries, the prevention and control measures are grossly inadequate, although the prevalence of the disease is very high [14, 15]. The lifestyle of the Mizan Aman town population is changing due to economic development, urbanization, and demographic transition. These rapid changes have led to the necessity of conducting a study on undiagnosed HTN and its associated factors at the community level. Further, many studies have been done at the facility level and focus on the overall prevalence of HTN. Therefore, this study is aimed to assess the prevalence of undiagnosed HTN and associated factors among adults in Mizan Aman town in the southwestern region of Ethiopia.

## 2. Methods

### 2.1. Study Setting, Design, and Period

A community-based cross-sectional study was conducted from April 1 to 30, 2021, in Mizan Aman town, the capital and administrative center of Bench Sheko Zone in the Southwestern Nations Nationalities Peoples Region (SWNNPR). Based on the report from the sub-city administration office, the total population of Mizan Aman town is estimated to be 54,951; of the total, 26,925 of them are male and the remaining 28,026 are female. Among the total population of 54,951 residing in the town, about 60.3% are estimated to be above 18 years old. In the study area, 1 teaching hospital, 1 HC, 9 medium private clinics, 5 drug stores, and 5 health posts (HPs) provided curative and preventive services through 287 healthcare workers (HCWs).

### 2.2. Sampling Techniques

**Figure 1:**
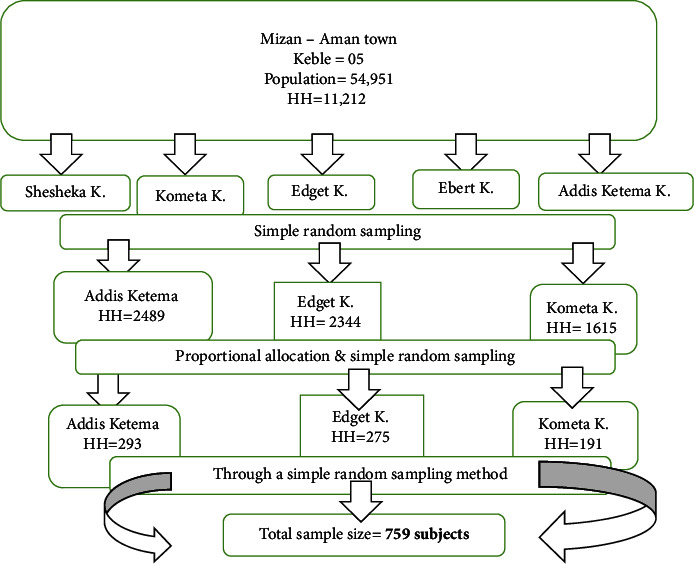
Schematic representation of a sampling procedure to assess the prevalence of undiagnosed HTN and associated factors among adult residents in Mizan Aman town, Bench Sheko Zone, Southwest Ethiopia, 2021.

A multistage sampling method was employed to recruit study participants. First, three kebeles (the smallest administration localities) (Addis Ketema, Edget, and Kometa Keble) were selected using simple random sampling from the five kebeles in the town. Second, households were allocated proportionally to each of the selected Keble and the households were selected within each kebele using the computer-generated random sampling technique (Figure 1). The sampling frame was prepared using the list of households from the family folder available at health posts. The frame is appropriate for this sampling since it contains the updated complete list of households within each of the kebeles considered for the study. Finally, one adult whose age was 18 years and above was selected from each household using a simple random sampling technique if there were two or more eligible adults living in the same household.

#### 2.2.1. Inclusion

Inclusion criteria included all adults aged 18 years and above who resided in the study area during the study period.

#### 2.2.2. Exclusion

Exclusion criteria included mentally ill patients, hospitalized patients, and medically confirmed cases of HTN since the objective of the study was on undiagnosed HTN only.

### 2.3. Sample Size Determination

The sample size was calculated for both specific objectives separately.

For the prevalence, the sample size was calculated using a single population proportion formula based on the following assumptions. The prevalence of undiagnosed HTN n Hawela Tula sub-city, Hawassa, southern Ethiopia (*p*=12.3%) was taken from a previous study [9]. For this study, 95% level of confidence, 3% margining of error (d), a design effect of 1.5 and 10% for a possible non-response rate was taken and the formula applied as shown below.

(1) $$\begin{array}{c} n=\frac{{\left(Z_{\left(a/2\right)}\right)}^{2}p\left(1-p\right)}{d^{2}}\text{.} \end{array}$$

Hence, the calculated sample size for the first specific objective becomes 759 subjects.

For the second objective, the sample size was calculated by using EPI INFO stat calc for population proportions to estimate the sufficient sample size. The variables considered in this estimation were being a male gender, family history of having HTN, Khat chewing, being in the age group of above 55 years, having BMI between 25 to 29.9, being in the age group from 35-54. From these variables, being a male gender yielded the maximum sample size of 238 with 16.13% of outcome in unexposed group, with an AOR of 2.5 at (1.2, 5.2) of CI, taking 95% confidence interval, 80% power, 1.5 a design effect and, 10% of non-response [9]. Hence, the calculated sample size for the second specific objective was 238 subjects.

Therefore, the determination for prevalence yielded the maximum number than the determination for factors and hence the final sample size for this study was found to be 759 subjects.

## 3. Data Collection

A structured and pretested questionnaire adapted from the WHO STEPwise approach for surveillance of NCDs in developing countries was used to interview the participants [16].

Data collection was done sequentially in a two-step process:

 
*Step 1*. Interview-based questionnaire on selected major health risk behaviors including smoking, alcohol consumption, poor fruit and vegetable consumption, and physical inactivity.
 
*Step 2*. Physical measures of health risks such as height, weight, blood pressure, body mass, and waist and hip circumference.

## 4. Data Collection Procedure

Using a pretested structured questionnaire, a face-to-face interview was conducted. After completing the interview, the physical measurements were conducted using the tape meter, digital automatic sphygmomanometer, and weight and height scales. We used calibrated instruments and standardized techniques to take the measurements.

Anthropometric measurements were taken based on the WHO guidelines, as specified in the Food and Nutrition Technical Assistance (FANTA) anthropometry manual [17]. Weight was measured to the nearest 0.1 kg with light clothes on a prestandardized digital body weighing scale, and height was measured to the nearest 0.1 cm in the standing position with no shoes, using a portable stadiometer, and BMI was calculated using the formula weight in kilogram divided by height in meters squared. Waist circumference was measured to the nearest 0.1 cm at the end of a normal expiration, at the midpoint between the lower part of the last rib and the top of the hipbone, using an inelastic measuring tape while the participants stood erect with their arms relaxed at the sides, and the hip circumference was a measure to the nearest 0.1 cm at the maximum circumference around the hips/maximum part of the buttocks. The waist circumference (cm) is divided by the hip circumference (cm) to estimate the waist-to-hip ratio (WHR). We take two measurements of weight, height waist, and hip circumference, and if the gap between the first two measurements was >0.1 kg for weight and >0.5 cm for height, hip, and waist circumference, we took the third measurement.

Blood pressure was measured in a sitting position with a supported back, and a digital automatic blood pressure (BP) device was used to measure the BP of the participants. The participants were taking rest for at least 5 min before measurement. Three measurements of BP on a single visit were taken at least one minute apart, and this survey considered the last two measures of BP levels and used their mean to detect HTN. At least two visits were made for those study participants whose BP was elevated at the first contact. According to the WHO guideline, a participant with systolic blood pressure (SBP) ≥140 mmHg or diastolic blood pressure (DBP) ≥90 mmHg will be diagnosed as an HTN case [18].

### 4.1. Data Quality Assurance

Data were collected by two senior nurses under one supervisor for each kebele following the training on interviewing techniques, anthropometric measurements, and handling of data collection instruments for one day. The questionnaire was prepared in English, then translated into Amharic, and then retranslated back to English to check its consistency. A pretest was done on 5% of the sample out of the study area and then an appropriate revision of the tool was done. Double-entry of the data to EpiData software for data verification was also performed.

### 4.2. Data Analysis

Data analysis was done by SPSS for Windows version 25, and the descriptive analysis was undertaken; the result was presented using frequency tables, graphs, and descriptive statistical summaries. The undiagnosed HTN status has a dichotomized response of yes and no, and hence bivariable analysis was performed using binary logistic regression to identify candidate variables for the multivariable logistic regression model to identify explanatory variables associated with the outcome variable. Then, those variables with a *p* value <0.25 were included in multivariable logistic regression for adjustment of confounding factors. Odds ratios (ORs) with 95% confidence intervals were calculated to measure the strength of association, and *p* value <0.05 was considered statistically significant. The Hosmer–Lemeshow test was used to assess the fitness of the model (chi-square: 3.9 with a *p* value of 0.9).

### 4.3. Operational Definition

Standard operational definitions were adapted for key variables to maintain consistency and uniformity of the information.

Undiagnosed HTN: adults (aged 18 years and above) will be considered undiagnosed for HTN if, at the time of the survey, he or she was diagnosed as hypertensive (SBP ≥140 mmHg or DBP ≥90 mmHg) but never took any prescribed antihypertensive medicine to lower or control blood pressure and was never been told by a health professional that they have HTN before this study [18].

Harmful use of alcohol: alcohol consumption of more than 14 units/week for men and more than 8 units/week for women in the last 12 months before the survey. Its calculation is then as follows: unit of alcohol = vol (in ml) X % alcohol/1000, and for different local alcoholic beverages, it is as follows: tella (4%), Tej (10%), and arake (40–45%) (glass: 250 ml and bottle: 330 ml) [19].

Low consumption of fruits and vegetables: less than 5 servings (<400 gm) of fruit and or vegetables per day in that 1 serving is defined as one orange/apple/banana or three tablespoons of cooked vegetables [16].

Current smoker: an adult who has smoked 100 cigarettes in his or her lifetime and who currently smokes cigarettes. Previous smoker: an adult who has smoked at least 100 cigarettes in his or her lifetime but who had quit smoking at the time of the interview. Non-smoker: an adult who has never smoked or who has smoked less than 100 cigarettes in his or her lifetime [20].

Physical activity: the subjects' physical activity was classified as high, moderate, and low.

High physical activity is defined as a vigorous-intensity activity of at least 3 days achieving a minimum total physical activity of at least 1500 min/week or 7 or more days of any combination of walking, moderate-intensity, or vigorous-intensity activities achieving a minimum total physical activity of at least 3000 min/week. Moderate physical activity is defined as 3 or more days of vigorous-intensity activity of at least 20 min per day or 5 or more days of moderate-intensity activity and/or walking of at least 30 min per day or 5 or more days of any combination of walking, moderate-intensity, or vigorous-intensity activities achieving a minimum total physical activity of at least 600 min/week. Low physical activity: not fulfilling the criteria for moderate and high physical activity [21].

A family history of HTN is considered if a person's first-degree relative (parent, grandparent, or sibling) had been diagnosed with HTN and/or received drug therapy for HTN [22].

## 5. Result

### 5.1. Sociodemographic Characteristics

A total of 759 participants were randomly selected and included in the study in Mizan Aman town with 97.2% response, and data were collected from 738 study subjects, 482 (65.3%) males and 256 (34.7%) females. The highest percentage (51.9%) of the study participants was in the age categories of 18–34 years, while 13% were in the age of 55 years and above. The mean age of the respondents was 38.85 (SD: 14.57) years (Table 1).

### 5.2. Behavioral Characteristics

The number of current smokers was 196 (26.6%) and all smoked manufactured tobacco products. The mean age when first started smoking was 26.4 (SD: 6.3). The majority (63.8%) of respondents declared that someone smoked at the workplace in their presence within the past seven days. The proportion of current and past drinkers was 30.3% and 30.6%, respectively. Among the current drinkers, 20.1% of them were men, but only 5.7% of women drank four or more days in the last week before the survey. About 62.3% of the study participants consumed more than five servings of fruit and vegetables per day for more than three days in a typical week. Among the study participants who responded about their physical activity, 22.1%, 19.1%, and 58.8% had low, moderate, and high levels of physical activity, respectively. Above one-fourth of the study subjects' body mass index (BMI) was greater than 25 kg/m^2^ (Table 2).

### 5.3. Prevalence of Undiagnosed HTN and Associated Risk Factors

#### 5.3.1. Prevalence of Undiagnosed HTN

The mean SBP and mean DBP of the study participants were 119.97 mmHg (SD: 17.4) and 80.48 mmHg (SD: 11.74), respectively. In the current study, the prevalence of undiagnosed HTN in Mizan-Aman was 14.8% [12.3–15.6] 95% CI. The prevalence of undiagnosed HTN among the study participants varied across their age, sex, serving fruit and vegetables, physical activity, and BMI. The prevalence of undiagnosed HTN was higher among older age participants (≥55 years and above age group) than the younger age group, and again the prevalence of undiagnosed HTN was higher among participants with higher BMI (25.2%) than normal BMI (11.9%). It was also higher among participants who had low physical activity (37.4%) compared with high physical activity (7.4%).

#### 5.3.2. Factors Associated with Undiagnosed HTN

Those in the age group of 55 years and above were 3.1 times higher at risk for undiagnosed HTN compared to the 18–34 years of age group (AOR = 3.138, 95% CI [1.511–6.516]). The odds of having undiagnosed HTN was 2.2 times higher in male than in female (AOR = 2.239, 95% CI [1.295–3.870]).

Low consumption of fruit and vegetables was positively associated with undiagnosed HTN. Those eating fruit and vegetables less than five servings per day for less than three days per week had 4.5 times (AOR = 4.549, 95% CI [2.352–8.800]) increased risk of undiagnosed HTN compared to those eating more than five servings per day for more than three days per week. Besides, the odds of having undiagnosed HTN were 3.9 times higher among those who did not take part in high physical activity compared to those who took part in high physical activity (AOR = 3.878, 95% CI [1.803–8.341]).

In addition, the odds of having undiagnosed HTN were 2.7 times higher among those whose BMI was greater than or equal to 25 kg/m^2^ when compared to those whose BMI was less than 25 kg/m^2^ (AOR = 2.667, 95% CI [1.551–4.588]) (Table 3).

## 6. Discussion

This community-based study has attempted to determine the prevalence, which is 14.8%, and identified factors associated with undiagnosed HTN. The high prevalence observed might be related to increasing urbanization, lack of awareness, and willingness to participate in regular health check-ups in the absence of health problems and barriers to screening services in Mizan Aman town.

Characteristics such as age, sex, physical activity, servings of fruit and vegetables, and BMI were predicted undiagnosed HTN.

The finding on the prevalence of undiagnosed HTN was in line with the findings of the study in the Gulele sub-city of Addis Ababa (13.3%), Hawassa (12.3%), in Ethiopia [7, 9]. This finding was closer to findings outside of Ethiopia, India (15.2%), and Bangladesh (11.1%) [13, 23]. The current finding was lower than the findings of studies conducted in a rural area of West Bengal (24.1%), Gadarif in eastern Sudan (33.5%), and in River Nile State, Sudan (38.2%) [15, 23, 24]. This difference might be due to the study setting and sample size in that their studies were conducted in rural communities and used large sample sizes attributable to the differences in lifestyle in a different setting. However, it is higher than the findings reported from a study done in the Gilgel Gibe area in Ethiopia (7.5%) [25]. These differences might be related to increasing urbanization, lack of awareness, and willingness to participate in regular health check-ups in the absence of health problems, coupled with accessibility, barriers to screening services, and differences in the study population used in various studies.

This study showed that undiagnosed HTN significantly increased as age increased. Those with age groups of 55 years and above were 3.1 times more likely to develop undiagnosed HTN as compared to the age group of 18–34 years. This finding sharply contrasted with a study conducted in Nepal, which showed that elderly patients (≥65 years of age) had a lower likelihood of being undiagnosed for HTN than patients aged 15–24 years [4]. But this finding is in line with the study findings conducted in Addis Ababa, Southwest Ethiopia, and Gimbi in Ethiopia [3, 26, 27]. The finding was also supported by studies conducted outside of Ethiopia, Bangladesh and South India, which revealed that the magnitude of HTN increased with the increment of age [27–31]. It is mostly related to the biological effect that increases arterial resistance due to age-related changes in the arterial wall and the thickening of the arterial wall or arteriosclerotic structural alterations and calcification in old age.

Regarding the gender difference, males are 2.2 times more likely to be undiagnosed for HTN compared to females. This finding was similar to a study done in Hawela Tula sub-city, Hawassa, that men were at 2.5 times higher risk of undiagnosed HTN than women [9]. Again, this study finding was supported by a study conducted in Southwest Ethiopia, which showed that men were at higher risk for undiagnosed HTN than their counterparts [26]. Other studies which were done in Southern Ethiopia, Nepal, and Southern Tanzania uncovered that HTN was significantly higher in males than in females [4, 32, 33]. However, some studies demonstrated that the odds of having HTN were higher in women [24, 27]. Thus, the significant difference might be due to the presence of coexisting risk factors and having a lower frequency of health facility visits trained in males and hormonal variation. That is, androgens increase blood pressure via the renin-angiotensin system (RAS), which promotes oxidative stress leading to the production of vasoconstrictor substances and a reduction in nitric oxide availability [34]. Other studies suggested that ovarian hormones, especially estrogen, may have the potential to keep blood pressure lower, as well as the cellular, biochemical, and molecular mechanisms by which sex hormones may modify the effects of HTN on the cardiovascular system [35].

The current study revealed that there is an association between undiagnosed HTN and infrequent consumption of fruit and vegetable. Those who consume fruits and/or vegetables less than five servings per day for three or fewer days in a typical week were 4.5 times more likely to develop undiagnosed HTN compared to those who consume fruits and/or vegetables for more than five servings per day for three or more days in a typical week. This finding was supported by a study done in the Gulele sub-city of Addis Ababa, which shows that the prevalence of undiagnosed HTN in people who did not consume fruits and vegetables in a typical week was 3 times more likely than those who consume fruits and vegetables 4–7 times in a typical week [7]. Studies conducted in Southwest Ethiopia also pointed out that eating fruit and vegetables three or fewer days per week was associated with undiagnosed HTN [26]. It is widely accepted that fruit and/or vegetables are considered an important component of a healthy diet and that their consumption could help prevent a wide range of CVDs including HTN CDs, and the World Health Organization (WHO) aims to promote this consumption [36].

The odds of having undiagnosed HTN were increased by 3.9 times among those who were not involved in high physical activity compared to those who did a high physical activity. This finding was concordant with a study conducted in Southwest Ethiopia and Nepal, which conveyed that those involved in vigorous activity were less likely to develop HTN than their counterpart [26, 37]. Hence, these findings suggest that several cardiometabolic problems may arise as a consequence of insufficient physical activity. Rapid urbanization, high population density, increased use of motorized vehicles, and modern technology might be predisposing factors for low physical activity among this study population.

The other important finding was about the association of BMI and undiagnosed HTN that being overweight or obese increases the risk of undiagnosed HTN. Those who had a BMI greater than or equal to 25 kg/m^2^ were about 2.7 times more likely at risk of undiagnosed HTN compared to those with a BMI of less than 25 kg/m^2^. This finding was similar to a study conducted in Jigjiga town of Ethiopia that those who had BMI ≥25 were 2 and 2.8 times more likely to be undiagnosed hypertensive when compared to those who had BMI less than 25, respectively [26, 38, 39]. Moreover, the current study agreed with different findings from Gondar city, Durame town, and Hawela Tula sub-city of Hawassa in Ethiopia, India, and Nigeria [9, 25, 40–42]. It could be related to urbanization, changes in dietary habits, and reduced physical activity that leads to obesity, while this study was quite lower than a study conducted in Hawassa that identified BMI was among the factors associated with HTN BMI 25 kg/m^2^ where 5 times more at risk for HTN than those with BMI less than 25 kg/m^2^ as well as in a hospital-based study in Bahir Dar Felege-Hiwot referral hospital that peoples of BMI ≥25 kg/m^2^ were 4.79 folds more likely to develop undiagnosed HTN than those whose BMI is less than 18.5 Kg/m^2^ (underweight) individuals [38]. Some of the variability between these reports could be due to the study setting and the difference in lifestyle. There are several mechanisms hypothesized to explain the link between obesity and HTN. It is generally thought that the accumulation of visceral and ectopic fat in several tissues and organs alters the metabolic and hemodynamic pathways, and additionally, insulin resistance and inflammation may promote an altered profile of vascular function and consequently lead to the development of HTN in obese people [43]. The reduction of overweight and obesity by improving nutrition and increasing regular physical activity is the best way to avoid or improve HTN [43]. It is important to teach people with high BMI to use interventions that could reduce their BMI and check their BP regularly to reduce the risk of undiagnosed HTN and its consequences.

This article was in preprint in Research Square since October 2022 [44].

## 7. Strengths and Limitations

To our knowledge, this is the first study to assess the prevalence of undiagnosed hypertension and its associated factors among adults in Mizan-Aman town, and the findings of this study provide a snapshot of the burden of hypertension among the population. Unlike other studies, this study was conducted at community level which can help interventions and future researchers to have more reliable findings. However, the WHO STEPwise protocol employed in this study involved gathering self-reported information on the participants' sociodemographic characteristics and information on participants' behavioral variables and hence might be subjective to respondent bias and readers might consider this limitation. Furthermore, this study lacked some important measurements like biochemicals and hence was unable to determine the level of blood glucose and/or cholesterol in the study.

## 8. Conclusion

This study revealed that the prevalence of undiagnosed HTN was high in Mizan-Aman town. It indicated that there might be a large number of people who have HTN but are not aware of it. Generally, it was observed that undiagnosed HTN existed in all age groups and both sexes, indicating the vulnerability of the whole population, not just a specific segment.

## 9. Recommendation

Community-based preventive approaches like lifestyle modification, increasing awareness, and strengthening routine screening for early detection at primary health service facilities bring a considerable change in undertaking the problem effectively.

## Figures and Tables

**Table 1:** Sociodemographic characteristics of the study participants in Mizan-Aman town, Bench Sheko Zone, Southwest Ethiopia, 2021.

Variable	Category	Frequency (*n* = 738)	
		Number	Percent (%)
Age (years)	18–34	383	51.9
	35–54	259	35.1
	>55	96	13.0

Sex	Male	482	65.3
	Female	256	34.7

Educational level	Primary education and less	334	45.3
	Secondary education	302	40.9
	College and above	102	13.8

Religion	Orthodox	364	49.3
	Protestant	318	43.1
	Muslim	56	7.6

Ethnicity	Bench	307	41.6
	Kaffa	266	36.0
	Amhara	94	12.7
	Others	71	9.6

Marital status	Single	220	29.8
	Married	434	58.8
	Divorced	61	8.3
	Widowed	23	3.1

Occupation status	Govt. employee	241	32.7
	NG employee	100	13.6
	Self-employed	308	41.7
	Others	89	12.1

Average monthly income (ETB)	<1644	473	64.2
	≥1644	264	35.8

Family history of HTN	Yes	143	19.4
	No	595	80.6

Family history of DM	Yes	195	26.4
	No	543	73.6

**Table 2:** Behavioral characteristics of the study participants in Mizan-Aman town, Bench Sheko Zone, Southwest Ethiopia, 2021.

Variable		Category	Frequency (*n* = 738)	
			Number	Percent (%)
Smoking habit		Current smoker	196	26.6
		Previous smoker	44	6.0

Ever consumed alcohol		Yes	22	30.6

Currently consumed alcohol		Yes	200	27.1

Eat saturated oil		Frequently	526	71.3

Eat fatty food		Frequently	241	32.7

Add salt to food		Frequently	622	84.3

Serving of fruit and vegetable		Less than five servings	278	37.7
		Five and more servings	460	62.3

Physical activity		High	434	58.8
		Moderate	141	19.1
		Low	163	22.1

Sedentary life		Yes	221	29.9

Chat chewing	Ever	Yes	209	28.3
	Current	Yes	178	24.1

BMI		<25 kg/m^2^	579	78.5
		≥25 kg/m^2^	159	21.5

History of raised blood glucose/diabetes		Yes	180	24.4

**Table 3:** Bivariable and multivariable logistic regression model showing associated factors with undiagnosed HTN among the study participants in Mizan-Aman town, Bench Sheko Zone, Southwest Ethiopia, 2021.

Variable	Category	Undiagnosed HTN	COR [95% CI]	AOR [95% CI]
		Num (%)		
Age (years)	18–34	60 (15.7)	1	
	35–54	22 (8.5)	2 (1.19–3.35)^*∗*^	1.5 (0.65–3.19)
	>55	27 (28.1)	0.5 (0.28–0.80)^*∗*^	**3.1 (1.51–6.51)** ^*∗∗*^

Sex	Male	60 (12.4)	1.7 (1.10–2.52)^*∗*^	**2.2 (1.29–3.87)** ^*∗∗*^
	Female	49 (19.1)	1	

Marital status	Single	23 (10.5)	1	
	Married	60 (13.8)	0.7 (0.43–1.21)^*∗*^	0.4 (0.09–1.55)
	Divorced	20 (32.8)	0.2 (0.12–0.47)^*∗*^	0.4 (0.09–1.39)
	Widowed	6 (26.1)	0.3 (0.12–0.92)^*∗*^	0.3 (0.07–1.07)

Occupation status	Govt. employee	40 (16.6)	0.9 (0.39–1.96)^*∗*^	1.1 (0.47–2.58)
	NG employee	25 (25.0)	0.5 (0.16–0.81)^*∗*^	0.7 (0.26–1.76)
	Self-employed	31 (10.1)	1.5 (0.32–1.45)^*∗*^	2.2 (0.94–5.26)
	Others	13 (14.6)	1	

Family history of HTN	Yes	29 (20.3)	0.6 (0.38–0.98)^*∗*^	0.6 (0.30–1.02)
	No	80 (13.4)	1	

Smoking habit	Current smoker	44 (22.6)	0.4 (0.28–0.67)^*∗*^	2 (0.78–5.03)
	Previous smoker	9 (20.5)	0.5 (0.22–1.07)^*∗*^	0.7 (0.26–1.88)
	Non-smoker	56 (11.2)	1	

Ever consumed alcohol	Yes	41 (19.6)	0.6 (0.39–0.92)^*∗*^	0.6 (0.34–1.17)
	No	68 (12.9)	1	

Serving of fruit and vegetable	Less than five servings	16 (5.8)	4.2 (2.38–7.22)^*∗*^	**4.5 (2.35–8.80)** ^*∗∗*^
	Five or more servings	93 (20.2)	1	

Eat saturated oil	Frequently	69 (13.1)	1.5 (1.00–2.36)^*∗*^	1.2 (0.69–2.03)
	Not frequently	40 (18.9)	1	

Eat fatty food	Frequently	28 (11.6)	1.5 (0.93–2.34)^*∗*^	1.5 (0.87–2.63)
	Not frequently	81 (16.3)	1	

Add salt to food	Frequently	86 (13.8)	1.5 (0.92–2.56)	1.5 (0.83–2.86)
	Not frequently	23 (19.8)	1	

Physical activity	High	61 (37.4)	1	
	Moderate	16 (11.3)	7.5 (4.65–12.1)^*∗*^	**7.3 (3.85–13.74)** ^*∗∗*^
	Low	32 (7.4)	4.7 (2.54–8.59)^*∗*^	**3.9 (1.80–8.34)** ^*∗∗*^

Sedentary life	Yes	51 (23.9)	0.4 (0.26–0.59)^*∗*^	0.7 (0.36–1.29)
	No	58 (11.0)	1	

History of diabetes	Yes	37 (21.1)	0.5 (0.35–084)^*∗*^	0.7 (0.38–1.25)
	No	72 (12.8)	1	

Ever chat chewing	Yes	52 (17.4)	0.7 (0.46–1.06)^*∗*^	0.8 (0.42–1.35)
	No	57 (13.0)	1	

BMI	Less than 25 kg/m^2^	69 (11.9)	1	
	Greater than 25 kg/m^2^	40 (25.2)	2.5 (1.60–3.85)^*∗*^	**2.7 (1.55–4.58)** ^*∗∗*^

^*∗*^Candidate variables in bivariable logistic regression at *p* value <0.25; ^*∗∗*^statistically significant variables in the final model of logistic regression at *p* value <0.05. Hosmer and Lemeshow's goodness-of-fit test produces a chi-square of 3.853 with a *p* value of 0.870.

## Data Availability

All data used during this study are available upon request from the corresponding author.

## Abbreviations

AOR: Adjusted odds ratio
BMI: Body mass index
CI: Confidence interval
DBP: Diastolic blood pressure
HTN: Hypertension
IRB: Institutional Review Board
MMHG: Millimeter of mercury
NCD: Non-communicable disease
OR: Odds ratio
SBP: Systolic blood pressure
SD: Standard deviation
SNNPR: South Nation Nationalities and Peoples Region
SPSS: Statistical Package for Social Sciences
WC: Waist circumference
WHO: World Health Organization
WHR: Waist-to-hip ratio.
